# Patterns of perceptions and beliefs about PrEP and their association with daily oral PrEP initiation among sexual and gender diverse adolescents in Brazil

**DOI:** 10.1590/0102-311XEN202725

**Published:** 2026-10-30

**Authors:** Suelen Seixas, Nila Galvão, Beo Oliveira Leite, Fabiane Soares, Leila Denise Alves Ferreira Amorim, Jony A. Pinto, Eliana Miura Zucchi, Dirceu B. Greco, Alexandre Grangeiro, Inês Dourado, Laio Magno

**Affiliations:** 1 Departamento de Ciências da Vida, Universidade do Estado da Bahia, Salvador, Brasil.; 2 Departamento de Ciências Exatas e da Terra, Universidade do Estado da Bahia, Salvador, Brasil.; 3 Instituto de Saúde Coletiva, Universidade Federal da Bahia, Salvador, Brasil.; 4 Departamento de Estatística, Universidade Federal da Bahia, Salvador, Brasil.; 5 Departamento de Estatística, Universidade Federal Fluminense, Niterói, Brasil.; 6 Universidade Católica de Santos, Santos, Brasil.; 7 Faculdade Israelita de Ciências da Saúde Albert Einstein, São Paulo, Brasil.; 8 Faculdade de Medicina, Universidade Federal de Minas Gerais, Belo Horizonte, Brasil.; 9 Faculdade de Medicina, Universidade de São Paulo, São Paulo, Brasil.; 10 Instituto Gonçalo Moniz, Fundação Oswaldo Cruz, Fiocruz, Salvador Brasil.

**Keywords:** Perception, Pre-Exposure Prophylaxis, Adolescent, Latent Class Analysis, Percepção, Profilaxia Pré-Exposição, Adolescente, Análises de Classes Latentes, Percepción, Profilaxis Pre-Exposición, Adolescente, Análisis de Clases Latentes

## Abstract

Perceptions and beliefs about pre-exposure prophylaxis (PrEP) for HIV are shaped by various factors and play a key role in its uptake. This study analyzed the relationship between these perceptions and PrEP initiation among adolescent men who have sex with men (AMSM) and adolescent transgender women (ATGW) in Brazil. This cross-sectional analysis used baseline data from the PrEP15-19 study, which evaluated the effectiveness of daily oral PrEP among AMSM and ATGW aged 15-19 years in three Brazilian cities from February 2019 to February 2023. Latent class analysis (LCA) was employed to identify patterns in PrEP-related perceptions and beliefs based on eight binary (agree/disagree) indicators. Logistic regression was used to estimate adjusted odds ratios (_adj_ORs) for the association between these patterns of perceptions and beliefs and PrEP initiation. A total of 1,477 adolescents were included in the analysis, the majority of whom were AMSM (91%) and aged 18-19 years (74.5%). Most participants identified as Black or Mixed-race (72%), and 81.4% initiated PrEP within 30 days of their initial study visit. LCA revealed two distinct groups based on perceptions and beliefs about PrEP: positive (n = 1,348; 93.2%) and negative (n = 99; 6.8%). Adolescents with positive perceptions and beliefs were significantly more likely to initiate PrEP (_adj_OR = 1.73; 95%CI: 1.05-2.84). Most participants held positive perceptions and beliefs about PrEP, which were strongly associated with timely initiation. These findings highlight the importance of addressing individual perceptions and beliefs and providing accurate, accessible information about PrEP. Educational interventions are essential to further improve PrEP awareness and uptake among vulnerable adolescent populations.

## Abstract (pt)

Percepções e crenças sobre a profilaxia pré-exposição (PrEP) ao HIV são moldadas por vários fatores. Elas desempenham um papel fundamental em sua adoção. Este estudo analisou a relação entre essas percepções e a iniciação da PrEP entre homens adolescentes que têm relações sexuais com homens (AHSH) e mulheres transgênero adolescentes no Brasil. Essa análise transversal utilizou dados de base do estudo PrEP15-19, que avaliou a eficácia da PrEP oral diária entre AHSH e mulheres transgênero adolescentes entre 15 e 19 anos em três cidades brasileiras de fevereiro de 2019 a fevereiro de 2023. Uma análise de classes latentes foi usada para identificar padrões em percepções e crenças relacionadas à PrEP com base em oito indicadores binários (concordar/discordar). Uma regressão logística foi usada para calcular razões de chance ajustadas (_aj_OR) acerca da associação desses padrões de percepção e a iniciação da PrEP. Este estudo incluiu 1.477 adolescentes, a maioria dos quais eram AHSH (91%) e com idades entre 18 e 19 anos (74,5%). A maioria se identificou como negra ou parda (72%) e 81,4% iniciou a PrEP dentro de 30 dias após a primeira consulta. A análise de classes latentes revelou dois grupos de percepção e crençasna PrEP: positivo (n = 1.348; 93,2%) e negativo (n = 99; 6,8%). Adolescentes com percepções e crenças positivas tiveram significativamente mais probabilidade de iniciar a PrEP (_aj_OR = 1,73; IC95%: 1,05-2,84). A maioria dos participantes tinha percepções e crenças positivas sobre a PrEP; essas estavam fortemente associadas à iniciação oportuna. Esses achados destacam a importância de abordar as percepções e crenças de indivíduos e fornecer informações precisas e acessíveis sobre a PrEP. Intervenções educacionais são essenciais para melhorar ainda mais a conscientização e a adoção da PrEP entre populações adolescentes vulneráveis.

## Abstract (es)

Las percepciones y creencias sobre la profilaxis previa a la exposición (PrEP) para el VIH están determinadas por diversos factores y desempeñan un papel clave en su adopción. Este estudio analizó la relación entre estas percepciones y el inicio de la PrEP entre adolescentes hombres que tienen relaciones sexuales con hombres (AHSH) y adolescentes transgénero mujeres en Brasil. Este análisis transversal utilizó datos de línea de base del estudio PrEP15-19,que evaluó la eficacia de la PrEP oral diaria entre AHSH y adolescentes transgénero mujeres de entre 15-19 años en tres ciudades brasileñas, desde febrero de 2019 hasta febrero de 2023. Se empleó el análisis de clases latentes (LCA) para identificar patrones en las percepciones y creencias relacionadas con la PrEP, basándose en ocho indicadores binarios (de acuerdo/en desacuerdo). Se utilizó la regresión logística para calcular las razones de probabilidades ajustadas (_aj_OR) delaasociación entre estos patrones de percepción y el inicio de la PrEP. Participaron un total de 1.477 adolescentes, la mayoría de los cuales eran AHSH (91%) y tenían entre 18-19 años (74,5%). La mayoría se identificó como de raza negra o morena (72%), y el 81,4% inició la PrEP dentro de los 30 días posteriores a su primera cita. El LCA reveló dos grupos distintos en cuanto a las percepciones y creencias sobre la PrEP: el grupo positivo (n = 1.348; 93,2%) y el grupo negativo (n = 99; 6,8%). Los adolescentes con percepciones y creencias positivas tenían una probabilidad significativamente mayor de iniciar la PrEP (_aj_OR = 1,73; IC95%: 1,05-2,84). La mayoría de los participantes tenían percepciones y creencias positivas sobre la PrEP, las cuales se asociaron estrechamente con el inicio oportuno del tratamiento. Estos hallazgos resaltan la importancia de abordar las percepciones y creencias individuales y de proporcionar información precisa y accesible sobre la PrEP. Las intervenciones educativas son esenciales para mejorar aún más el conocimiento y la adopción dela PrEP entre las poblaciones de adolescentes vulnerables.

## Introduction

Daily oral pre-exposure prophylaxis (PrEP) for HIV involves the consistent use of antiretroviral medications to prevent HIV infection before potential exposure ^1^ ^,^ ^2^. In Brazil, PrEP was incorporated into the Brazilian Unified National Health System (SUS, acronym in Portuguese) in late 2017 for individuals aged 18 and older. In 2022, national guidelines were updated to expand PrEP eligibility to adolescents aged 15 years and above, representing an important advance in HIV prevention policy for younger populations ^3^.

Despite these advances, an increase in HIV infection rates among adolescents and young people in Brazil has been observed, especially among populations of sexual and gender diversity ^4^ ^,^ ^5^ ^,^ ^6^. These groups remain disproportionately affected by structural vulnerabilities, stigma, discrimination, and social exclusion, factors that continue to limit access to and effective use of HIV prevention technologies, including PrEP ^7^ ^,^ ^8^ ^,^ ^9^. Adolescence is a period of significant social, biological, and psychological changes that can strongly influence health-related decision-making, particularly in contexts of greater vulnerability and inequality ^10^.

The decision to initiate PrEP may be shaped by various individual factors, including perceived risk of HIV infection, age, race, and sexual behavior patterns such as number of partners ^11^ ^,^ ^12^ ^,^ ^13^, as well as personal beliefs and attitudes toward PrEP use ^14^. Perceptions and beliefs about PrEP can directly influence preventive health behaviors and play a pivotal role in decisions to initiate use. Perception refers to the way individuals interpret and make sense of the world through sensory and cognitive processes, shaped by prior experiences and personal beliefs. This interpretive process does not always align with scientific evidence and may lead individuals to adopt or reject certain health behaviors, including PrEP use ^15^.

In this context, perceptions and beliefs should not be understood merely as individual cognitive constructs, but as elements that can influence the effectiveness of HIV prevention strategies. Misconceptions, fears related to side effects, concerns about social image, stigma, and moral judgments associated with PrEP use may compromise PrEP adherence ^16^. Conversely, positive beliefs grounded in accurate information about PrEP can promote adolescents’ engagement in prevention programs and enhance the impact of biomedical interventions ^17^. For sexual and gender diverse adolescents, whose interactions with health services are often shaped by experiences of stigma and exclusion, addressing perceptions and beliefs is essential for the development of effective prevention strategies ^18^ ^,^ ^19^.

Different dimensions of perceptions related to PrEP influence adolescents’ intention to initiate PrEP in distinct ways. Understanding how sexual and gender diverse adolescents perceive and believe in PrEP is essential to deepen our insight into how these perceptions and beliefs influence shared attitudes and, ultimately, the decision to use this HIV prevention method. Therefore, this study aimed to examine the association between patterns of perceptions and beliefs about PrEP and its initiation among adolescent men who have sex with men (AMSM) and adolescent transgender women (ATGW) in three Brazilian cities.

## Methods

### Study design

This cross-sectional study analyzed baseline data from the PrEP15-19 cohort study, collected between February 2019 and February 2023. The study enrolled AMSM and ATGW aged 15-19 years from three Brazilian cities: Belo Horizonte (Minas Gerais State), Salvador (Bahia State), and São Paulo. The main objectives of PrEP15-19 included evaluating the effectiveness of PrEP and its potential contribution to reducing HIV incidence in the target population. Upon study entry, eligible participants could choose to initiate PrEP (PrEP arm) or to use other combination prevention measures without PrEP (non-PrEP arm). The detailed methodology of the PrEP15-19 study is described in Dourado et al. ^20^.

Demand creation strategies included face-to-face and online approaches, using peer educators, social networking applications ^21^, and an artificial intelligence chatbot, “Amanda Selfie”, designed to represent a transgender woman ^22^. Researchers collected social-behavioral data through structured questionnaires administered by interviewers at enrollment and repeated quarterly thereafter. The questionnaire covered topics such as PrEP acceptability and knowledge, sociodemographic characteristics, sexual behaviors, use of prevention methods, substance use, history of sexually transmitted infections (STIs), and experiences of discrimination and violence. The study procedures were informed by formative qualitative research conducted within the PrEP15-19 study, which assessed the acceptability of daily oral PrEP among adolescent men who have sex with men, travestis, and transgender women in Brazil. Further details on this formative component are provided in Zucchi et al. ^23^. Trained health professionals entered all responses into an online data platform. Each interview had an average duration of 40 minutes.

All participants provided written informed consent. In São Paulo, minors under 18 were exempt from the parental consent requirement; in Salvador, this exemption applied only to those with broken family ties; and in Belo Horizonte, parental consent was required for all participants. Detailed descriptions of these ethical procedures are available in previous publications ^24^ ^,^ ^25^. All participants were informed of their right to withdraw from the study at any time and to decline to answer any questions they considered sensitive.

During the COVID-19 pandemic, the study sites followed local guidelines that included quarantine, social distancing measures, reduced public transportation, and the suspension of non-essential health services. However, contingency measures such as telemonitoring were implemented to ensure the continued provision of prevention services ^26^.

### Study variables

To construct the exposure variable “Patterns of perceptions and beliefs about PrEP”, the latent class analysis (LCA) method was used ^27^. Eight questionnaire items were used as indicators of perceptions and beliefs about PrEP. Response options included “agree”, “disagree”, “don’t know”, and “prefer not to answer”. We coded the latter two as missing. For analysis, each item was treated as a binary variable (i.e., agree and disagree) (Box 1). Missing responses were handled within the LCA framework using full-information maximum-likelihood estimation, allowing participants with partially missing indicators to remain in the analytical sample.

**Box 1:** Items used as indicators for the latent variable “Patterns of perceptions and beliefs about pre-exposure prophylaxis (PrEP)”.

1. PrEP is intended only for homosexual and transgender people
2. PrEP is meant only for individuals with many partners
3. PrEP is the same medication used for the treatment of HIV
4. PrEP can interfere with other medication, such as hormones and contraceptives
5. PrEP gives a negative image of those who use it
6. PrEP has many side effects
7. It is concerning that PrEP uses the same medication used to treat people living with HIV
8. It is uncomfortable to take preventive medication

The primary outcome was PrEP initiation, defined as the first PrEP dispensation within 30 days of the initial study visit (i.e., yes; no). Other covariates included: (i) sociodemographic: subpopulation (AMSM; ATGW), age (15-17 years; 18-19 years), race/color (White; Black or Mixed-race), education level (elementary; high school; higher education), study site (Salvador; São Paulo; Belo Horizonte); marital status (single; in a relationship), living arrangement (living with parents or relatives; not living with parents or relatives), and occupational status (working; without working), participation in a community-based organization or LGBTQIA+ NGO (no; yes), (ii) behavioral: age at first sexual intercourse (< 15 years; ≥ 15 years); number of casual partners (no partners; 1 or 2 partners; ≥ 3); perceived risk of HIV infection was originally measured on a scale from 1 to 10 and recoded into three categories: low risk (1-2), moderate risk (3-6), and high risk (7-10).

### Data analysis

LCA is an analytical technique that generates latent classes based on data patterns to identify groups of individuals according to their relationship with a theoretical construct of interest ^27^. LCA was employed to identify distinct patterns of perceptions and beliefs about PrEP. Parameter estimation for the LCA models used the Full Information Maximum Likelihood (FIML) method, which uses all available information while accounting for missing data in the indicators, assuming the data were Missing at Random (MAR) ^28^. LCA models with two to five classes were tested, with model selection based on the Bayesian information criterion (BIC), the sample-size adjusted BIC, and the Akaike information criterion (AIC). Entropy and interpretability of classes were also considered ^27^ ^,^ ^29^. The resulting latent variable represented different patterns of PrEP-related perceptions and beliefs. LCA was conducted using Mplus version 8.9 software (https://www.statmodel.com/).

Descriptive analyses characterized the sociodemographic and behavioral profiles, as well as the study population’s PrEP perceptions and beliefs. Bivariate analyses used chi-square tests to examine associations between covariates and both PrEP initiation and patterns of perceptions and beliefs about PrEP. A logistic regression model was then fitted using the “classify-analyze” approach (i.e., a naïve method), with latent class membership assigned by maximum probability as the main exposure variable. Crude odds ratios (OR) and adjusted OR (_adj_OR) 95% confidence intervals (95%CI) for the association between perceptions and beliefs about PrEP and its initiation were estimated, adjusting for potential confounders selected based on theoretical relevance or statistical significance (p < 0.10) in bivariate analyses with either the exposure or the outcome. The full model included all such covariates (initial model), and the final model retained those that either had theoretical importance or whose removal altered the _crude_OR by 10% or more (final model). Descriptive and inferential analyses were performed using STATA version 15.0 (https://www.stata.com).

### Ethical approval and consent to participate

The PrEP15-19 study was approved by the Ethics Research Committees of the University of São Paulo (n. 70798017.3.0000.0065), the Federal University of Bahia (n. 01691718.1.0000.5030), and the Federal University of Minas Gerais (n. 17750313.0.0000.5149), in accordance with *Resolution n. 466/2012* of the Brazilian National Health Council.

## Results

Among the 1,621 adolescents enrolled during the study period, 1,447 were included in the final analysis after excluding those who tested HIV-positive and those who had missing data for all items of the questionnaire used as indicators in the LCA model (Figure 1). Overall, participants were predominantly AMSM (91.0%; 1,317/1,447), aged 18-19 years (74.5%; 1,078/1,447), and self-identified as Black or Mixed-race (71.9%; 1,008/1,447). Most reported multiple casual sexual partners (39.4%; 563/1,447) and perceived themselves at moderate risk of HIV infection (52.4%; 754/1,447). Most initiated PrEP within 30 days of their first appointment (81.4%; 1,178/1,447). PrEP initiation was highest in São Paulo (85.2%; 624/732), among individuals with more than three sexual partners (86.1%; 485/563), and among those who perceived themselves at high risk for HIV infection (87.2%; 192/220) (Table 1).

**Figure 1:**
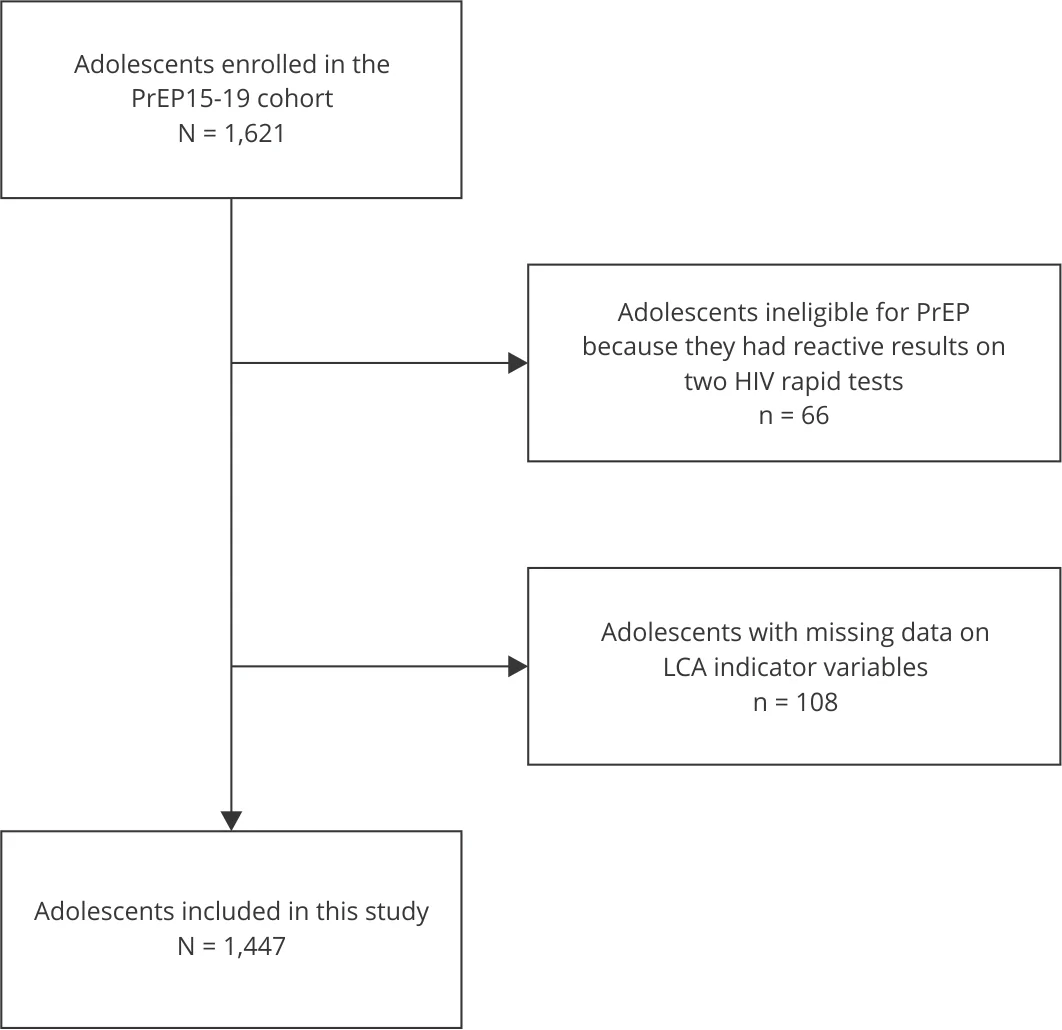
Flowchart of baseline enrollment and analytic sample selection in the PrEP15-19 cohort. Brazil, February/2019-February/2023.

LCA: latent class analysis; PrEP: pre-exposure prophylaxis.

**Table 1:** Sociodemographic and behavioral characteristics of adolescent men who have sex with men (AMSM) and transgender women (ATGW), and their distributions according to pre-exposure prophylaxis (PrEP) initiation. PrEP15-19 study, Brazil, 2019-2023.

Variables	Total population [N = 1,447]	PrEP initiation n (%)		p-value
	n (%)	Yes [1,178 (81.4)]	No [269 (18.5)]	
Sociodemographic				
Subpopulation				
AMSM	1,317 (91.2)	1,074 (81.5)	243 (18.4)	
ATGW	130 (8.9)	104 (80.0)	26 (20.0)	0.665
Age group (years)				
15-17	369 (25.5)	299 (81.0)	70 (18.9)	
18-19	1,078 (74.5)	879 (81.5)	199 (18.46)	0.828
Race/skin color				
White	393 (28.0)	321 (81.6)	72 (18.3)	
Black or Mixed-race	1,008 (71.9)	821 (81.4)	187 (18.5)	0.920
Education level				
Elementary school	102 (7.0)	79 (77.4)	23 (22.5)	
High school	1,029 (71.2)	846 (82.2)	183 (17.7)	
Higher education	314 (21.7)	252 (80.2)	62 (19.7)	0.411
Study site				
Salvador	434 (29.9)	327 (75.3)	107 (24.6)	
São Paulo	732 (50.5)	624 (85.2)	108 (14.7)	
Belo Horizonte	281 (19.4)	227 (80.7)	54 (19.2)	< 0.001
Marital status				
Single	926 (64.3)	764 (82.5)	162 (17.4)	
In a relationship	512 (35.6)	406 (79.3)	106 (20.7)	0.135
Living arrangement				
With parents or relatives	1,191 (82.3)	968 (81.2)	223 (18.7)	
Not residing with parents or relatives	256 (17.6)	210 (82.0)	46 (17.9)	0.778
Occupational status				
Working	826 (57.1)	662 (80.1)	164 (19.8)	
Without working	621 (42.9)	516 (83.0)	105 (16.9)	0.154
Participation in a community-based organization or LGBTQIA+ NGO				
Yes	150 (10.3)	119 (79.3)	31 (20.6)	
No	1,294 (89.6)	1,056 (81.6)	238 (18.3)	0.498
Behavioral				
Age at first sexual intercourse (years)				
< 15	628 (44.4)	520 (82.8)	108 (17.2)	
≥ 15	784 (55.5)	634 (80.8)	150 (19.1)	0.350
Number of casual partners				
No	433 (30.3)	331 (76.4)	102 (23.5)	
1 or 2	430 (30.1)	344 (80.0)	86 (20.0)	
≥ 3	563 (39.4)	485 (86.1)	78 (13.8)	< 0.001
Perceived risk of HIV infection				
Low	463 (32.2)	367 (79.2)	96 (20.7)	
Moderate	754 (52.4)	612 (81.1)	142 (18.8)	
High	220 (15.3)	192 (87.2)	28 (12.7)	0.040

NGO: nongovernmental organizations.

To measure and describe the latent variable “perceptions and beliefs about the use of PrEP”, a series of LCA models ranging from two to five classes were compared, with parameter estimates and parsimony evaluated to select the best model. The diagnostic criteria pointed to the 2- and 3-class models as most appropriate; however, the interpretability of the latent classes supported the final 2-class model (Table 2).

**Table 2:** Comparison between the classes of the latent class analysis (LCA) models, according to goodness of fit indices and entropy measure. PrEP15-19 study, Brazil, 2019-2023.

Criteria for model selection	2 classes	3 classes	4 classes	5 classes
AIC	7742.301	7731.196	7722.164	7725.378
BIC	7832.014	7868.405	7906.868	7957.577
BIC-sample-size adjusted	7778.011	7785.811	7795.684	7817.803
Entropy	0.765	0.817	0.568	0.590

AIC: Akaike information criterion; BIC: Bayesian information criterion

Table 3 presents the overall frequency of agreement with each statement and the conditional probabilities by latent class. Overall, agreement with statements reflecting negative perceptions and beliefs about PrEP was relatively low in the study population. The most frequently endorsed statements were that PrEP is the same medication used for HIV treatment (33.2%; 331/997), has many side effects (26%; 212/814), and may harm the user’s social image (24.6%; 332/1,348). Lower frequencies were observed for beliefs that PrEP is intended only for homosexual and transgender individuals (14.1%; 189/1,338), is appropriate only for individuals with multiple partners (11.3%; 158/1,393), is uncomfortable to use (10.7%; 147/1,376), may interact with other medications (9.4%; 66/701), and it is an inconvenience that PrEP is the same as HIV treatment (8.7%; 108/1,237).

**Table 3:** Description of latent patterns of perceptions and beliefs about pre-exposure prophylaxis (PrEP) among adolescent men who have sex with men (AMSM) and transgender women (ATGW), obtained by latent class analysis (LCA). PrEP15-19 study, Brazil, 2019-2023.

Indicators (n/N) *	Frequency (%) **	Conditional probabilities (%) **	
		Positive perceptions and beliefs [n = 1,348; 93.2%]	Negative perceptions and beliefs [n = 99; 6.8%]
PrEP is intended only for homosexual and transgender people (189/1,338)	14.1	12.9	24.3
PrEP is meant only for individuals with many partners (158/1,393)	11.3	9.3	30.0
PrEP is the same medication used for the treatment of HIV (331/997)	33.2	30.4	56.7
PrEP can interfere with other medications, such as hormones and contraceptives (66/701)	9.4	6.6	38.2
PrEP can give a negative image of those who use it (332/1,348)	24.6	21.1	57.7
PrEP has many side effects (212/814)	26.0	20.3	73.4
It is concerning that PrEP uses the same medication used to treat people living with HIV (108/1,237)	8.7	4.6	46.0
It is uncomfortable to take preventive medication (147/1,376)	10.7	5.6	58.5

Note: bold values indicate conditional probabilities at least 25% higher or lower than the corresponding overall frequency in the study population.

* Denotes the number of “agree” responses over the total number of valid responses for each indicator;

** Frequencies (%) and conditional probabilities (%) correspond to the “agree” response category for each indicator.

Based on the selected LCA model, two distinct classes were identified, representing positive and negative perceptions and beliefs about PrEP. The positive perceptions and beliefs class (n = 1,348; 93.2%) was characterized by consistently low probabilities of agreement with unfavorable or inaccurate statements about PrEP. In contrast, the negative perception class (n = 99; 6.8%) showed substantially higher probabilities of agreement, particularly regarding concerns about side effects (73.4%), discomfort in taking preventive medication (58.5%), fear of negative social image (57.7%), and the belief that PrEP is the same medication used for HIV treatment (56.7%) (Table 3).

Bivariate analyses showed variation in the distribution of perception and belief classes across selected sociodemographic characteristics. Positive perceptions and beliefs were more frequent among AMSM, adolescents aged 18-19 years, those who had completed high school, those who were not working, and those who did not participate in community-based organizations or LGBTQIA+ NGOs (Table 4).

**Table 4:** Bivariate analysis of sociodemographic and behavioral covariates among adolescent men who have sex with men (AMSM) and transgender women (ATGW) and by patterns of perceptions and beliefs about pre-exposure prophylaxis (PrEP). PrEP15-19 study, Brazil, 2019-2023.

Variables	Negative [n (%)]	Positive [n (%)]	p-value
Sociodemographic			
Subpopulation			
AMSM	80 (6.0)	1,237 (93.9)	
ATGW	19 (14.6)	111 (85.3)	< 0.001
Age group (years)			
15-17	32 (8.6)	337 (91.3)	
18-19	67 (6.2)	1,011 (93.7)	0.107
Race/Skin color			
White	21 (5.3)	372 (94.6)	
Black or Mixed-race	73 (7.2)	935 (92.7)	0.202
Education level			
Elementary school	18 (17.6)	84 (82.3)	
High school	60 (5.8)	969 (94.1)	
Higher education	21 (6.6)	293 (93.3)	< 0.001
Study site			
Salvador	32 (7.3)	402 (92.6)	
São Paulo	49 (6.6)	683 (93.3)	
Belo Horizonte	18 (6.4)	263 (93.5)	0.860
Marital status			
Single	62 (6,7)	864 (93.3)	
In a relationship	36 (7.0)	476 (92.9)	0.809
Living arrangements			
With parents or relatives	74 (6.2)	1,117 (93.7)	
Not residing with parents or relatives	25 (9.7)	231 (90.2)	0.041
Occupational status			
Working	71 (8.6)	755 (91.4)	
Without working	28 (4.5)	593 (95.4)	0.002
Participation in a community-based organization or LGBTQIA+ NGO			
Yes	19 (12.6)	131 (87.3)	
No	80 (6.1)	1,214 (93.8)	0.003
Behavioral			
Age at first sexual intercourse (years)			
< 15	48 (7.6)	580 (92.3)	
≥ 15	43 (5.4)	741 (94.5)	0.101
Number of casual partners			
No	34 (7.8)	399 (92.1)	
1 or 2	27 (6.2)	403 (93.7)	0.581
≥ 3	36 (6.3)	527 (93.6)	
Perceived risk of HIV infection			
Low	33 (7.1)	430 (92.8)	
Moderate	45 (5.9)	709 (94.0)	0.353
High	19 (8.6)	201 (91.3)	

NGO: nongovernmental organizations.

Multivariate logistic regression analyses showed that adolescents classified in the positive perceptions and beliefs about PrEP group had higher odds of initiating PrEP compared with those in the negative group: in the initial model, _adj_OR = 1.71 (95%CI: 1.03-2.81); in the final model, _adj_OR = 1.73 (95%CI: 1.05-2.84) (Table 5).

**Table 5:** Odds ratios (OR) and 95% confidence intervals (95%CI) for the association between perceptions and beliefs about pre-exposure prophylaxis (PrEP) and its initiation among adolescent men who have sex with men (AMSM) and transgender women (ATGW). PrEP15-19 study, Brazil, 2019-2023**.**

Variables	_crude_ **OR (95%CI)**	Model 1 *	Model 2 **
		_adj_ **OR (95%CI)**	_adj_ **OR(95%CI)**
Perceptions and beliefs about PrEP			
Negative	1.00	1.00	1.00
Positive	1.78 (1.10-2.89)	1.71 (1.03-2.81)	1.73 (1.05-2.84)
Subpopulation			
AMSM	-	1.00	-
ATGW	-	1.07 (0.63-1.79)	-
Age group (years)			
15-17	-	1.00	-
18-19	-	1.06 (0.75-1.51)	-
Race/Skin color			
White	-	1.00	-
Black or Mixed-race	-	1.16 (0.84-1.62)	-
Education level			
Elementary school	-	1.00	1.00
High school	-	1.06 (0.62-1.83)	1.12 (0.67-1.89)
Higher education	-	0.85 (0.45-1.58)	0.89 (0.50-1.57)
Study site			
Salvador	-	1.00	1.00
São Paulo	-	1.92 (1.38-2.68)	1.85 (1.35-2.53)
Belo Horizonte	-	1.39 (0.94-2.06)	1.35 (0.92- 1.98)
Living arrangements			
With parents or relatives	-	1.00	-
Not residing with parents or relatives	-	1.03 (0.70-1.53)	-
Occupational status			
Working	-	1.00	-
Without working	-	0.86 (0.64-1.15)	-
Participation in a community-based organization or LGBTQIA+ NGO			
No	-	1.00	-
Yes	-	0.95 (0.60-1.51)	-
Number of partners			
No	-	1.00	1.00
1 or 2	-	1.21 (0.87-1.69)	1.22 (0.87-1.70)
≥ 3	-	2.01 (1.42-2.84)	2.00 (1.42-2.84)
Perceived risk of HIV infection			
Low	-	1.00	1.00
Moderate	-	0.98 (0.72-1.33)	0.98 (0.73-1.34)
High	-	1.44 (0.89-2.34)	1.46 (0.90-2.36)

NGO: nongovernmental organizations.

* Adjusted (adj) for: subpopulation, age group, race, education level, study site, living arrangements occupational status, participation in NGOs, number of partners, and perceived risk of HIV infection (N = 1,369);

** Adjusted for: education level, study site, number of partners, and perceived risk of HIV infection (N = 1,369).

## Discussion

LCA identified two distinct patterns of perceptions and beliefs about PrEP among AMSM and ATGW adolescents: positive and negative. Most participants belonged to the positive perceptions and beliefs class, which was associated with PrEP initiation. This reflects exposure to the PrEP15-19 project’s outreach strategies efforts via social media, dating apps, school-based education, and community-based PrEP promotion ^21^.

Adolescents classified in the positive perceptions and beliefs class were less likely to endorse concerns about possible interactions with hormones and contraceptives, discomfort related to the continuous use of preventive medication, and the fact that PrEP uses the same medication as HIV treatment. These results indicate that medication-related concerns may play an important role in shaping sexual and gender diverse adolescents perceptions and beliefs about PrEP ^30^ ^,^ ^31^.

Despite the overall positive outlook, some negative perceptions and beliefs persist, particularly stigma-related concerns, such as the fear that using PrEP could result in a negative social image ^13^. Studies conducted in France ^32^ and the United States ^33^ have similarly documented concerns about stigma and social judgment related to PrEP use. In the French study, approximately one-third of participants reported fear of negative social perceptions.

Compared with studies among adult gay and bisexual men in Australia, where positive perceptions of PrEP were twice as prevalent as negative ones and were associated with reduced HIV-related anxiety ^34^. In our study, some adolescents exhibited persistent negative beliefs, indicating developmental and contextual differences that may limit the applicability of adult-focused PrEP messaging strategies.

From a Health Belief Model perspective, adolescents in the positive perceptions and beliefs class reported lower perceived barriers, such as concerns about side effects or the burden of continuous medication use, and greater perceived benefits, which facilitate PrEP initiation. In contrast, stigma-related beliefs acted as relevant perceived barriers, particularly when PrEP use is associated with moral judgments or stigmatized social identities ^35^ ^,^ ^36^.

Importantly, some adolescents perceived PrEP as a prevention strategy intended exclusively for AMSM or ATGW populations. While PrEP has been prioritized for key populations given their disproportionate vulnerability to HIV, this perception should be interpreted with caution, given that the study included only AMSM and ATGW participants. In addition, during most of the PrEP15-19 Phase 1 follow-up period, Brazilian PrEP guidelines primarily emphasized key populations, and PrEP recommendations for the general population expanded only after the 2022 update of the Brazilian Clinical Protocol and Therapeutic Guidelines (PCDT, acronym in Portuguese) ^3^. These factors may have reinforced the perception of PrEP as a strategy restricted to specific groups. Nevertheless, such misconceptions may also reflect internalized stigma or fear of disclosure, further discouraging engagement with PrEP services ^35^ ^,^ ^37^.

A considerable proportion of participants perceived PrEP as a prevention strategy primarily intended for individuals with multiple sexual partners. Such perceptions may contribute to limiting PrEP acceptability among adolescents who do not identify with this profile. Shame and anticipated stigma when seeking care can further limit PrEP uptake, even among those at risk ^38^.

Overall, fostering positive perceptions and beliefs of PrEP among adolescents requires interventions that go beyond information provision to address stigma, social norms, and developmental vulnerabilities. Aligning PrEP delivery and communication strategies with adolescents’ lived experiences and social and cultural contexts is essential to promote timely PrEP initiation, strengthen combination HIV prevention efforts, and ultimately improve adolescents’ sexual health outcomes.

### Limitations

This study has several limitations. First, its cross-sectional design, based on baseline data from a cohort study, precludes causal inference regarding the relationship between perceptions and beliefs about PrEP and PrEP initiation. Selection bias may be present, as adolescents engaged in PrEP-related services or research initiatives may already hold more favorable perceptions of PrEP. Information bias is also possible due to the use of self-reported data and the potential influence of social desirability. Although the questionnaire was administered at baseline, participants may have been exposed to PrEP-related information through social networks, community initiatives, or informal sources before their first visit. Second, the structured questionnaire developed for the PrEP15-19 project, while designed to be accessible to adolescents, may not fully capture culturally specific meanings of PrEP use and stigma. Additionally, latent class membership was assigned using the maximum posterior probability and subsequently included in the regression model through a classify-analyze approach. Although commonly used, this approach may introduce classification error and biased estimates when relating latent classes to distal outcomes. Future studies could consider three-step or bias-adjusted approaches to account for classification uncertainty ^39^ ^,^ ^40^ ^,^ ^41^. Finally, the broader social and cultural context in which adolescents form their beliefs, including family norms, stigma, and misinformation, should be considered when interpreting these findings.

## Conclusions

This study found that while most adolescents held positive perceptions and beliefs about PrEP, a minority expressed negative perceptions and beliefs that could hinder its uptake. Positive perceptions and beliefs were significantly associated with PrEP initiation. Addressing persistent misconceptions and social stigma is critical for expanding access to PrEP. Culturally informed educational strategies that consider adolescents’ beliefs and experiences are necessary to improve PrEP awareness and use. The scarcity of data on adolescents’ perceptions and beliefs regarding PrEP in Brazil highlights the need for further research.

## Data Availability

The research data are available upon request to the corresponding author.
